# Gestational and Lactational Atrazine Exposure Potentially Mediates Behavioral and Dopaminergic Alterations in Rat Offspring: Insights into Nurr1-Related ceRNA Regulation

**DOI:** 10.3390/ijms27093818

**Published:** 2026-04-25

**Authors:** Yongjie Ma, Tianao Sun, Minglian Pan, Zhanyue Zheng, Jingxia Wei, Xinyu Yuan, Jinhao Wan, Yingjie Zhou, Yan Sun

**Affiliations:** 1 School of Public Health, Guilin Medical University, Guilin 541199, China; mayongjie0606@163.com (Y.M.); 17791635922@163.com (T.S.); pml18278456264@163.com (M.P.); zhengzhanyue999@163.com (Z.Z.); m18777893571@163.com (J.W.); 18285094298@163.com (X.Y.); wjhwio@163.com (J.W.); 15221582965@163.com (Y.Z.); 2 Guangxi Key Laboratory of Environmental Exposomics and Entire Lifecycle Health, Guilin Medical University, Guilin 541199, China

**Keywords:** atrazine, dopaminergic neurons, Parkinson’s disease, ceRNA network, *Nurr1*, neuroinflammation

## Abstract

This study aimed to investigate the molecular mechanisms underlying dopaminergic injury induced by gestational and lactational atrazine (ATR) exposure in rat offspring, with a particular focus on non-coding RNA-mediated regulation. Pregnant rats were exposed to ATR during gestation and lactation. Offspring underwent behavioral testing at postnatal day 21 (PND21) and were sacrificed for midbrain tissue collection at PND28. Behavioral alterations, histopathological changes in the substantia nigra, and dopaminergic marker expression were assessed to evaluate ATR-induced neurotoxicity. Whole-transcriptome sequencing was then performed to identify differentially expressed mRNAs, miRNAs, and lncRNAs, followed by co-expression, protein–protein interaction, and competing endogenous RNA (ceRNA) network analyses. Key targets were validated by qRT-PCR. Candidate molecules identified from transcriptomic and ceRNA analyses were further examined in an ATR-induced neurotoxicity model established in RA-differentiated *SK-N-SH* cells. Dual-luciferase reporter, Ago2-RNA immunoprecipitation, and biotin-labeled RNA pull-down assays were used to examine putative binding relationships and molecular interactions. In addition, lentivirus-mediated *Elavl4* overexpression was performed to further evaluate the role of this candidate regulator in ATR-induced Nurr1 downregulation. Gestational and lactational ATR exposure induced significant behavioral abnormalities in rat offspring. These changes were accompanied by histopathological alterations in the substantia nigra, including reduced *TH* immunoreactivity, as well as abnormal expression of dopaminergic markers, characterized by decreased *TH* and *Nurr1* levels and increased α-syn expression. Together, these findings indicate the presence of dopaminergic injury. Whole-transcriptome analysis further revealed widespread dysregulation of mRNAs, miRNAs, and lncRNAs in ATR-exposed offspring. Subsequent integrative analysis suggested a potential ceRNA regulatory relationship among *Elavl4*, *miR-301a-5p*, and *Nurr1*, which was further supported by qRT-PCR. Dual-luciferase reporter, RIP, and RNA pull-down assays supported direct interactions between *miR-301a-5p* and both *Elavl4* and *Nurr1*, as well as their association with the Ago2-containing silencing complex. Moreover, Elavl4 overexpression partially reversed ATR-induced Nurr1 downregulation in vitro. Gestational and lactational ATR exposure induced behavioral abnormalities and dopaminergic injury in rat offspring. Whole-transcriptome analysis combined with experimental validation suggests a potential association between the *Elavl4/miR-301a-5p/Nurr1* ceRNA axis and ATR-induced dopaminergic injury, providing insight into the post-transcriptional mechanisms underlying developmental neurotoxicity.

## 1. Introduction

Neurodegenerative diseases have shown a sustained rise in prevalence in recent decades, emerging as a major global public health challenge. Among them, disorders involving dopaminergic neuronal dysfunction—most notably Parkinson’s disease (PD)—are characterized by progressive motor deficits accompanied by cognitive decline and non-motor disturbances. These symptoms severely impair patients’ quality of life and impose increasing social and economic burdens as global aging accelerates [1,2]. Although multiple therapeutic agents such as levodopa and dopamine receptor agonists are available, these interventions primarily offer symptomatic relief. They neither prevent nor reverse dopaminergic neuronal degeneration and, with prolonged use, may result in debilitating motor complications [3]. Consequently, elucidating the mechanisms underlying dopaminergic neurodegeneration and identifying potential molecular targets represent urgent priorities.

Mounting evidence highlights the crucial role of environmental factors in modulating dopaminergic neuronal vulnerability. Atrazine, one of the most heavily used triazine herbicides worldwide, is of particular concern due to its environmental persistence and tendency to accumulate in water bodies and food chains [4,5]. Beyond its well-recognized endocrine-disrupting effects, atrazine has been shown to interfere with central nervous system development, triggering behavioral abnormalities and neurochemical dysregulation across multiple species [6]. Early-life exposure appears especially critical, as the developing nervous system is highly sensitive to external insults. Animal studies demonstrate that atrazine exposure during developmental windows can induce long-lasting neurobehavioral deficits and alterations in neuronal circuitry [7]. Yet, despite these findings, the molecular mechanisms by which atrazine contributes to dopaminergic neuronal injury remain inadequately understood.

Non-coding RNAs, including microRNAs (miRNAs) and long non-coding RNAs (lncRNAs), have recently emerged as key regulators of neurotoxicity induced by environmental chemicals. Evidence suggests that dysregulated ncRNAs can mediate neuronal degeneration through pathways related to autophagy, inflammation, oxidative stress, and post-transcriptional modulation [8]. However, how these ncRNAs integrate into the broader transcriptomic landscape of atrazine-induced dopaminergic injury has not been systematically investigated. Additionally, epidemiological and molecular data linking atrazine exposure—particularly during sensitive early developmental periods—to increased risk of neurodegenerative disease later in life remain limited [9], highlighting a major knowledge gap.

Advances in high-throughput sequencing and systems biology have provided powerful means to decipher complex neurotoxic mechanisms. Transcriptome-wide analyses combined with co-expression network modeling enable comprehensive identification of gene regulatory relationships, uncovering central molecular nodes and pathways involved in toxicant-induced neurodegeneration [10,11]. Such integrative approaches are essential for understanding how environmental chemicals perturb coordinated gene networks rather than isolated molecular targets.

Against this background, the present study aimed to uncover the molecular mechanisms underlying dopaminergic neurotoxicity induced by early-life atrazine exposure. By integrating high-throughput RNA sequencing with co-expression and competing endogenous RNA (ceRNA) network construction, we systematically mapped the regulatory architecture associated with atrazine-induced neuronal dysfunction. Our findings provide new insights into how environmental toxicants perturb neurodevelopment through transcriptional and post-transcriptional regulatory pathways and offer a theoretical foundation for early prevention and targeted intervention strategies for neurodegenerative disorders.

To substantiate this hypothesis at the molecular level, we performed comprehensive validation experiments in a cellular model. The Dual-Luciferase Reporter Assay confirmed sequence-specific interactions among the three components. RNA immunoprecipitation (RIP) assays further demonstrated their co-existence within the functional RNA-induced silencing complex (RISC). In addition, RNA pull-down assays provided direct evidence of their physical interaction. Collectively, these findings establish a solid experimental foundation for elucidating the post-transcriptional regulatory mechanisms underlying ATR-induced neurotoxicity.

## 2. Results

### 2.1. General Condition and Body Weight Changes in Rats

**Figure 1:**
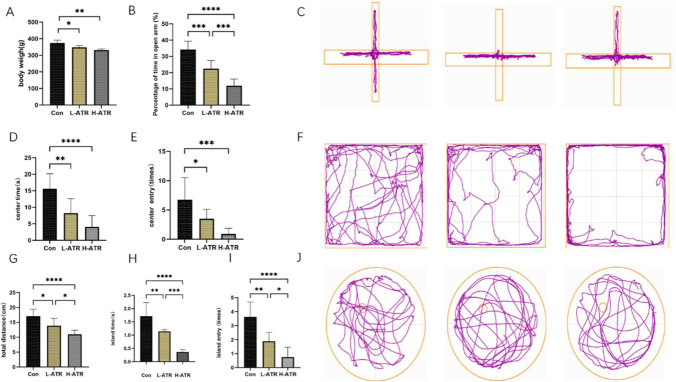
(**A**): Mouse body weight comparison; (**B**): Percentage of open arm time in elevated plus maze; (**C**): Elevated plus maze trajectory map; (**D**): Central area dwell time in open field test; (**E**): Number of central area crossings in open field test; (**F**): Open field test trajectory map. (**G**): Total Distance Traveled in Water Maze Exploration, (**H**): Time Spent on Platform in Water Maze, (**I**): Number of Platform Crossings in Water Maze, (**J**): Water Maze Trajectory Diagram (*n* = 8, * *p* < 0.05, ** *p* < 0.01, *** *p* < 0.001, **** *p* < 0.0001, One-way ANOVA followed by Tukey’s post hoc test).

Body weight was used as the primary indicator of general health status. Daily weight measurements were recorded, and comparisons were made at gestational day 19 (Figure 1A). Both the low-dose ATR group (L-ATR) and the high-dose ATR group (H-ATR) showed significantly reduced body weight relative to the control group (*p* < 0.01). This reduction is consistent with characteristic phenotypes observed in Parkinson’s disease (PD) animal models.

### 2.2. Atrazine-Induced Behavioral Alterations

To evaluate locomotor and cognitive function, multiple behavioral tests were conducted:

Elevated Plus Maze (EPM): ATR-exposed offspring exhibited a significantly lower percentage of time spent in the open arms compared with controls (*p* < 0.05), indicating an increase in anxiety-like behavior. The H-ATR group showed a more pronounced effect (Figure 1B,C).

Open Field Test (OFT): ATR-exposed rats displayed reduced center time and fewer center crossings (*p* < 0.05), along with diminished locomotor activity and heightened anxiety-like behavior. Again, the H-ATR group demonstrated stronger behavioral impairments (Figure 1D–F).

Morris Water Maze (MWM): In the probe trial, ATR exposure significantly decreased platform residence time, total distance traveled, and platform crossings compared to controls (*p* < 0.05), indicating impaired spatial memory (Figure 1G–J). The H-ATR group showed more severe deficits than the L-ATR group.

Collectively, these findings demonstrate that early-life atrazine exposure successfully induced PD-like motor and non-motor deficits, confirming its in vivo neurotoxic effects.

### 2.3. Validation of Dopaminergic System Alterations and Pathological Injury

**Figure 2:**
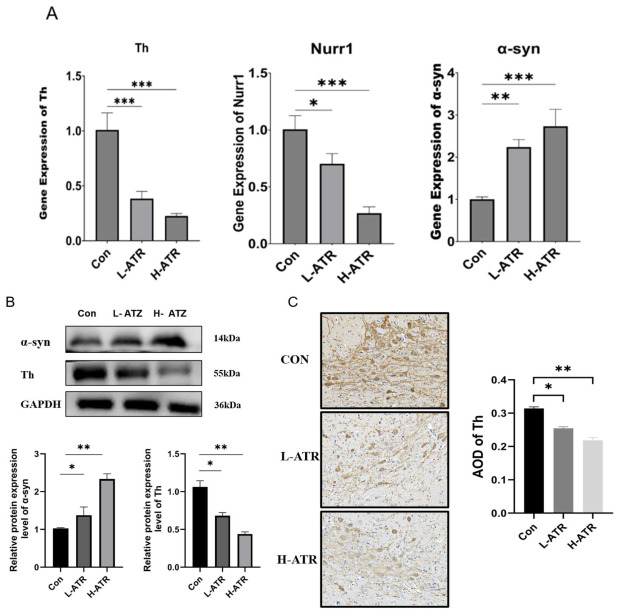
(**A**): The relative mRNA expression levels of key dopaminergic markers (*Th*, *Nurr1*, and *α-syn*) in the substantia nigra of rats from the Con, L-ATR, and H-ATR groups, as determined by qRT-PCR; (**B**): Representative Western blot images and corresponding quantitative densitometric analysis of *Th* and *α-syn* protein expression levels; (**C**): Representative immunohistochemical (IHC) staining images showing the progressive loss of *Th*-positive dopaminergic neurons in the substantia nigra, alongside the quantitative analysis of the Average Optical Density (AOD) of *Th*. The magnification or scale of the microscope is the same, 400 times. Data are presented as the mean ± SD (n = 3 independent biological replicates per group). (* *p* < 0.05, ** *p* < 0.01, and *** *p* < 0.001, One-way ANOVA followed by Tukey’s post hoc test).

To validate the transcriptomic findings and assess the extent of ATR-induced neurotoxicity, we evaluated the expression of key dopaminergic markers in the substantia nigra at both the mRNA and protein levels. QRT-PCR analysis revealed that ATR exposure induced a significant, dose-dependent downregulation of *Th* and *Nurr1* mRNA, alongside a marked upregulation of *α-syn* mRNA (Figure 2A).

Crucially, to provide definitive pathological evidence, we further performed Western blot and immunohistochemical (IHC) analyses. Consistent with the transcriptional changes, Western blotting demonstrated a substantial dose-dependent decrease in Th protein levels and a concomitant accumulation of α-syn protein in ATR-exposed rats (Figure 2B). To further characterize the loss of dopaminergic integrity, we examined the morphological changes via IHC staining for Th in the midbrain (Figure 2C). In the control group, Th-positive neurons exhibited a robust distribution with deep brown staining and intricate dendritic networks. However, following ATR exposure, a progressive loss of immunoreactivity was observed. Specifically, in the H-ATR group, Th-positive cells appeared markedly sparse and shrunken, with significantly diminished staining intensity. Quantitative assessment of the Average Optical Density (AOD) corroborated these morphological and mRNA findings, showing a significant decline from 0.31 in the control group to 0.25 in the L-ATR group (*p* < 0.05), and further dropping to 0.21 in the H-ATR group (*p* < 0.01). Together, these robust expression patterns—specifically the progressive loss of Th and the aberrant accumulation of α-syn—provide compelling physical evidence for the successful induction of dopaminergic neuronal injury in this model, closely mirroring key features of Parkinsonian-like neurotoxicity.

### 2.4. Differential Gene Screening

**Figure 3:**
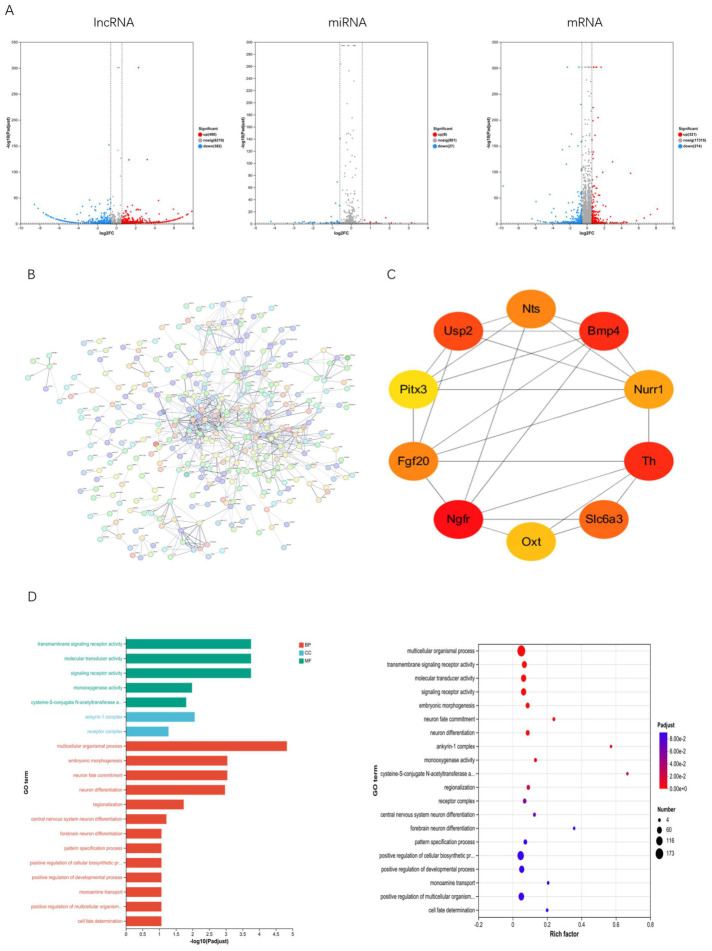

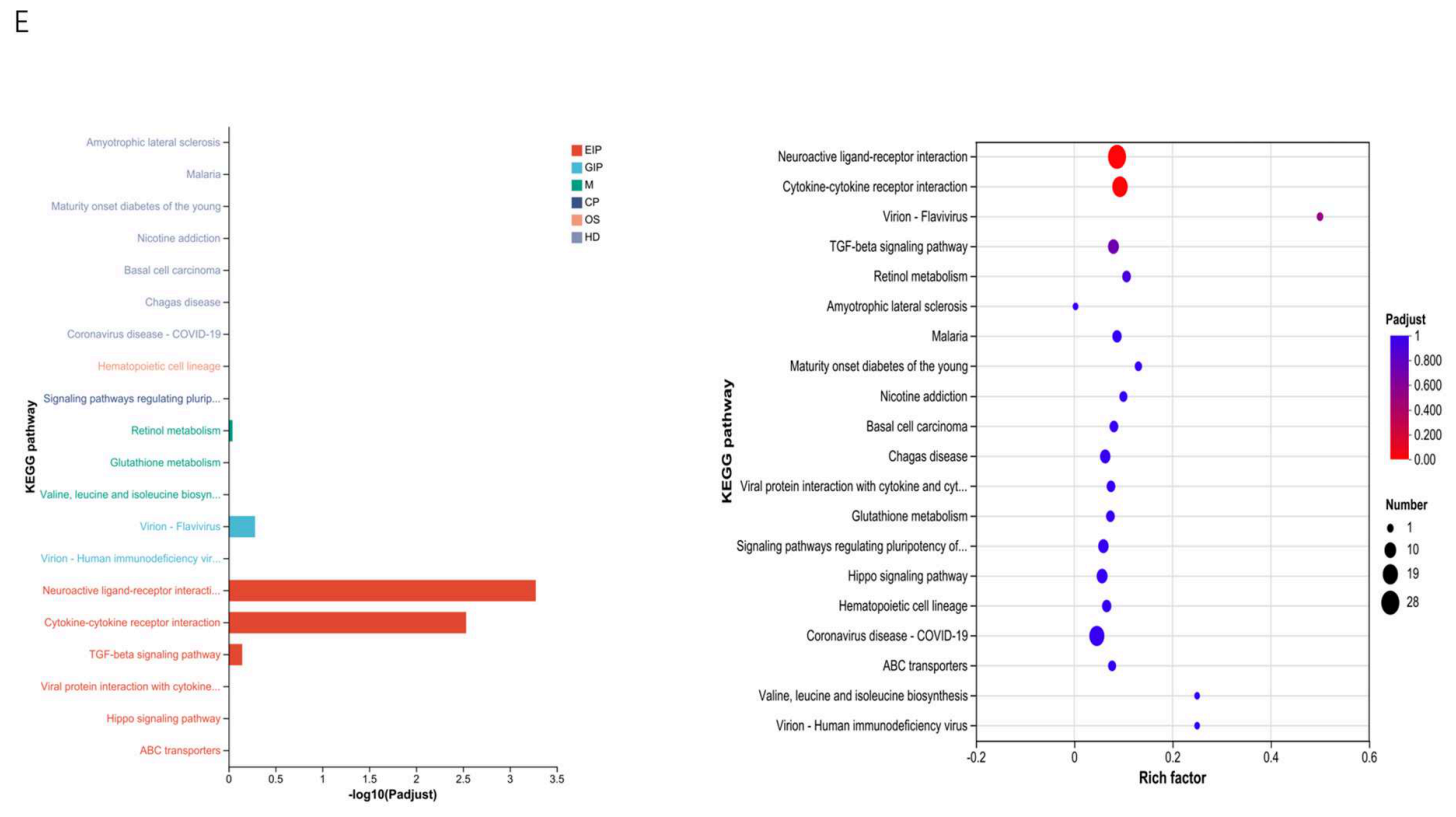
(**A**): Volcano plot of differentially expressed genes, (**B**): Protein–protein interaction (PPI) network, (**C**): Top 10 hub genes, (**D**): Gene Ontology (GO) functional enrichment analysis of differentially expressed mRNAs, (**E**): KEGG functional enrichment analysis of differentially expressed mRNAs.

To elucidate the molecular mechanisms underlying the aforementioned behavioral phenotypes, we performed whole-transcriptome sequencing on rat brain tissue. Based on the whole-transcriptome analysis, three full gene expression datasets were obtained (lncRNA; miRNA; mRNA). As shown in the volcano plot (Figure 3A), differentially expressed genes were visualized as follows: red indicates upregulated genes with log_2_FC > 0.5 and *p* < 0.05; blue indicates downregulated genes with log_2_FC < −0.5 and *p* < 0.05; gray represents genes with non-significant differences. A total of 862 DEGs were identified for lncRNA (480 upregulated, 382 downregulated), 35 DELs for miRNA (8 up, 27 down), and 595 DEMs for mRNA (321 up, 274 down).

### 2.5. PPI Network Construction and Hub Gene Screening

To identify the most functionally core genes among hundreds of differentially expressed mRNAs, we uploaded 595 differentially expressed mRNA genes to STRING for PPI network construction, selecting rat as the organism. The PPI data were downloaded into Cytoscape_v3.10.0 software to construct a comprehensive PPI network (Figure 3B). Subsequently, the CytoHubba plugin screened the top 10 genes using four algorithms. The top-scoring hub genes were identified as *Bmp4*, *Th*, *Nurr1*, *Fgf20*, *Slc6a3*, *Usp2*, *Nts*, *Oxt*, *Pitx3*, and *Ngfr*. Subsequently, using the Cytoscape plugin “cytoHubba,” we designed an interaction network among the 10 hub DEGs based on their participation levels (Figure 3C).

### 2.6. Enrichment Analysis

GO analysis revealed significant enrichment of differentially expressed mRNAs across 927 entries. The top 20 most enriched entries are displayed (Figure 3D), including multicellular processes, embryonic morphogenesis, and neuronal fate specification under Biological Process (BP); anchor protein complexes and receptor complexes under Cellular Component (CC); and signal receptor activity and molecular transduction activity under Molecular Function (MF). KEGG analysis revealed significant enrichment of genes in the Neuroactive Ligand-Receptor Interactions and Cytokine-Cytokine Receptor Interactions pathways (Figure 3E). This suggests these pathways may play crucial roles in PD pathogenesis through mechanisms such as regulating neurotransmitter receptors, neuroinflammation, and the gut–brain axis.

### 2.7. Non-Coding RNA Regulatory Networks Reveal Upstream Regulation of Key Dopamine Genes

**Figure 4:**
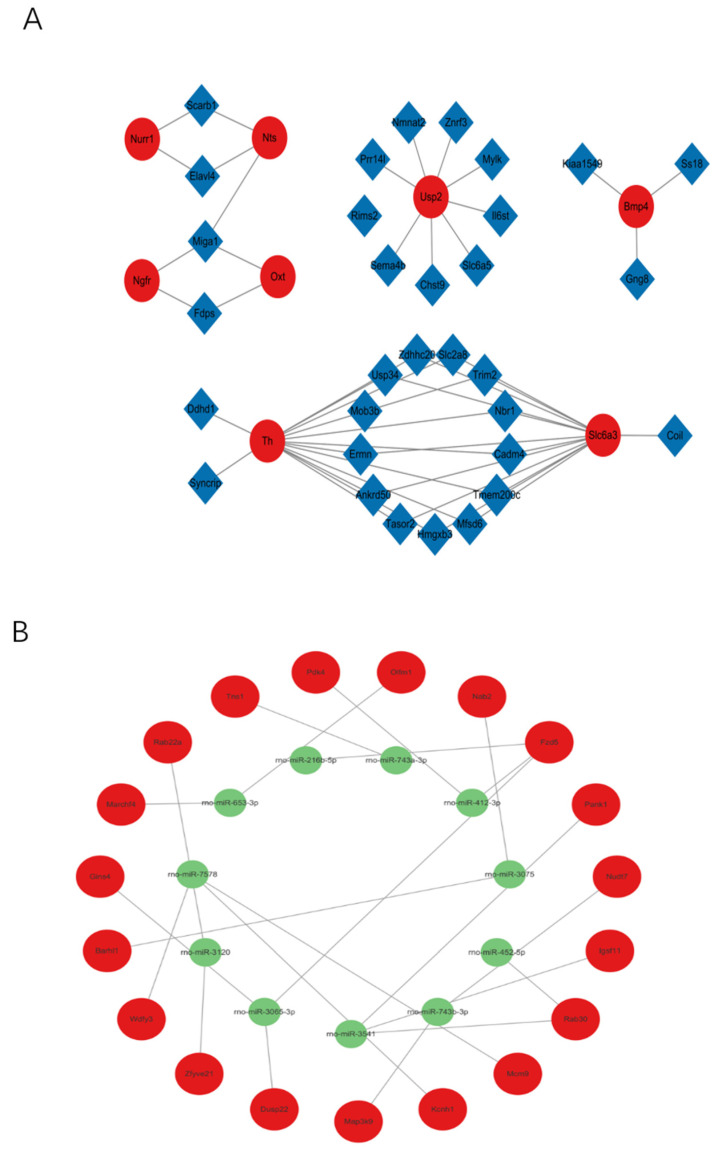

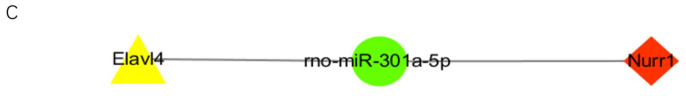
(**A**): lncRNA-mRNA network, (**B**): miRNA-mRNA network, (**C**): endogenous RNA network. Yellow triangles represent lncRNAs, green circles represent miRNAs, and red diamonds represent mRNAs.

Given the multitude of differentially expressed transcripts, we adopted a hierarchical screening strategy to elucidate the upstream regulatory mechanisms. First, *Nurr1* was selected as the focal anchor from our top 10 hub genes. While other hub genes like *Th* and *Slc6a3* exhibited robust dysregulation, they primarily function as downstream structural or enzymatic effectors. In contrast, *Nurr1* acts as an upstream master transcription factor directly responsible for driving the expression of *Th* and *Slc6a3* and is essential for the survival and maintenance of dopaminergic neurons. Therefore, anchoring our search on *Nurr1* provided the highest probability of identifying an upstream epigenetic regulatory network (ceRNA) that could comprehensively explain the widespread downstream dopaminergic collapse. Co-expression networks of lncRNA-mRNA and miRNA-mRNA were then constructed using databases such as Starbase and encase (Figure 4A,B). Focusing on *Nurr1*, we utilized computational prediction tools (TargetScan, miRWalk) to identify upstream miRNAs targeting *Nurr1*, leading to the identification of rno-miR-301a-5p. Finally, we cross-referenced our sequencing data to identify co-downregulated lncRNAs harboring binding sites for this shared miRNA. This unique intersection isolated lncRNA *Elavl4* as the prime candidate, thereby establishing a specific ceRNA regulatory axis: *Elavl4*–miR-301a-5p–*Nurr1* (Figure 4C).

### 2.8. qRT-PCR Validation

**Figure 5:**
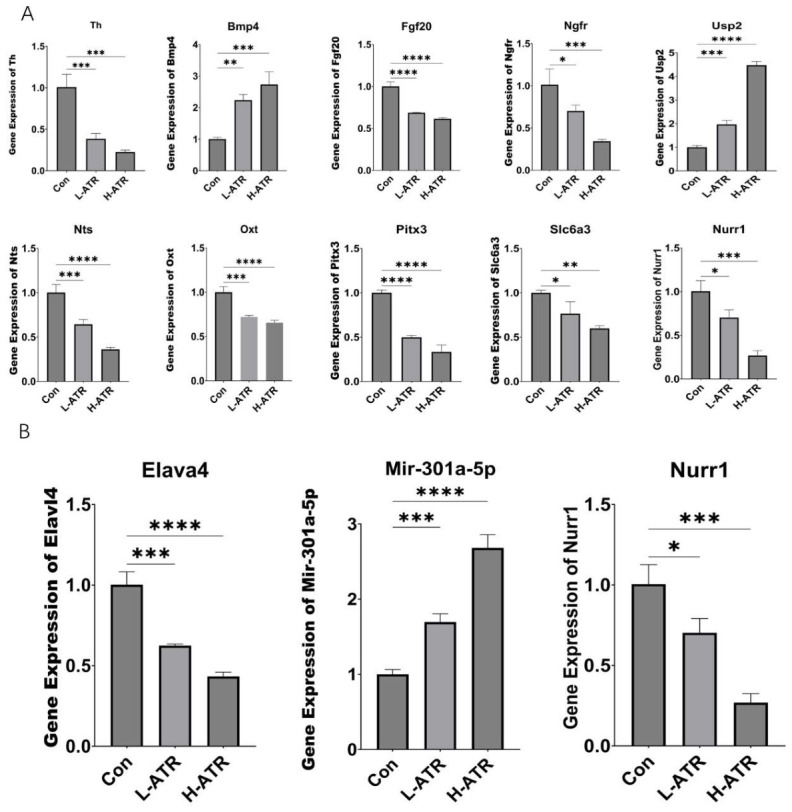
(**A**): PCR results for the top 10 hub genes; (**B**): PCR results for lncRNA, miRNA, and mRNA. (* *p* < 0.05, ** *p* < 0.01, *** *p* < 0.001, **** *p* < 0.0001, One-way ANOVA followed by Tukey’s post hoc test).

qRT-PCR validation was performed on the top 10 hub genes (Figure 5A) and critical lncRNA–miRNA–mRNA components (Figure 5B). The expression trends were highly consistent with sequencing data, confirming the robustness and reliability of the transcriptomic analyses.

Prior to in vitro validation, bioinformatic sequence alignment confirmed that the predicted miR-301a-5p binding seed sequences within the 3′UTRs of both *Nurr1* and *Elavl4* are highly conserved between rat and human. This strong evolutionary conservation provided a robust rationale for utilizing the differentiated human SK-N-SH cell line to evaluate the proposed post-transcriptional interactions.

### 2.9. Dual-Luciferase Reporter Assay Confirms Direct Targeting of Elavl4 and Nurr1 by miR-301a-5p

**Figure 6:**
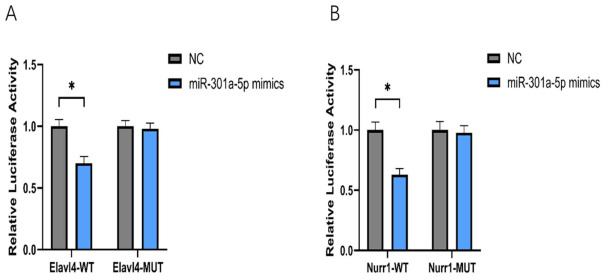
(**A**): *miR-301a-5p* Directly Targets *Nurr1*; (**B**): *miR-301a-5p* Directly Binds to *Elavl4*. (* *p* < 0.05, One-way ANOVA followed by Tukey’s post hoc test).

The results demonstrated that, compared with the NC group, co-transfection with *miR-301a-5p* significantly decreased the relative luciferase activity of the *Nurr1* wild-type (*Nurr1*-WT) reporter construct by approximately 50% (*p* < 0.01). In contrast, mutation of the predicted binding site within the *Nurr1* 3′UTR (*Nurr1*-MUT) completely abolished the inhibitory effect of miR-301a-5p (Figure 6A). These findings confirm that *Nurr1* is a direct downstream target of miR-301a-5p.

Similarly, miR-301a-5p markedly suppressed the luciferase activity of the *Elavl4* wild-type (*Elavl4*-WT) reporter construct, whereas no significant effect was observed in the *Elavl4* mutant (*Elavl4*-MUT) construct (Figure 6B). This result indicates that the lncRNA *Elavl4* harbors specific binding elements for miR-301a-5p, providing the structural and functional basis for its role as a competing endogenous RNA (ceRNA) or molecular sponge.

### 2.10. Ago2-RIP Assay Reveals Functional Co-Existence of Elavl4, miR-301a-5p, and Nurr1 Within the RISC

**Figure 7:**
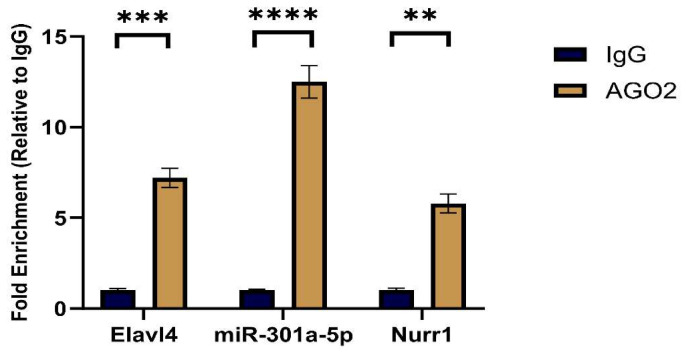
Results of the RNA Immunoprecipitation (RIP) Assay. (** *p* < 0.01, *** *p* < 0.001, **** *p* < 0.0001, One-way ANOVA followed by Tukey’s post hoc test).

Compared with the IgG control group, *Elavl4*, *miR-301a-5p*, and *Nurr1* were significantly enriched in the Ago2 immunoprecipitated fraction, with enrichment levels exceeding 5-fold (*p* < 0.01) (Figure 7).

These results indicate that, in dopaminergic neuron-like cells, endogenous *Elavl4* and *Nurr1* are physically associated with the Ago2 protein, and that *miR-301a-5p* is also present within the same complex. This provides direct cellular evidence that *Elavl4* and *Nurr1* may competitively bind *miR-301a-5p* in an Ago2-dependent manner, thereby supporting the proposed ceRNA regulatory mechanism.

### 2.11. RNA Pull-Down Assay Confirms the Direct Physical Interaction Between Elavl4 and miR-301a-5p

**Figure 8:**
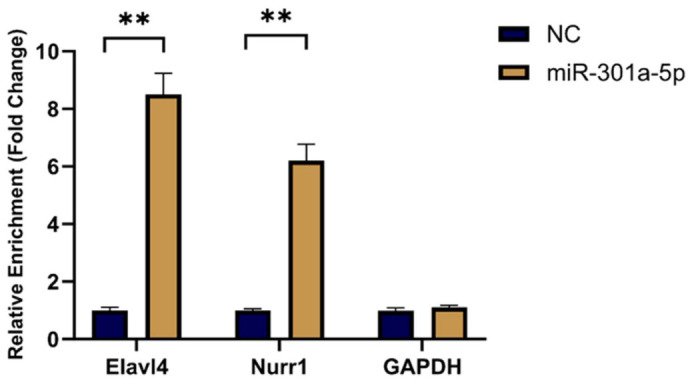
RNA Pull-Down Analysis Confirms the Direct Interaction Between *Elavl4* and *miR-301a-5p*. (** *p* < 0.01, One-way ANOVA followed by Tukey’s post hoc test).

Following transfection with biotin-labeled *miR-301a-5p* (Bio-*miR-301a-5p*), streptavidin-mediated pull-down assays revealed significant enrichment of *Elavl4* and *Nurr1* transcripts in the captured complexes compared with the Bio-NC control group (*p* < 0.01) (Figure 8), as determined by qRT-PCR analysis.

In reciprocal experiments, biotin-labeled *Elavl4* probes (Bio-*Elavl4*) specifically enriched *miR-301a-5p*, further confirming their direct interaction.

Collectively, these data provide direct biochemical evidence that *Elavl4* physically associates with *miR-301a-5p*, thereby sequestering it and potentially reducing its suppressive effect on the downstream target gene *Nurr1*.

### 2.12. Lentivirus-Mediated Overexpression of Elavl4 Rescues ATR-Induced Suppression of Nurr1

**Figure 9:**
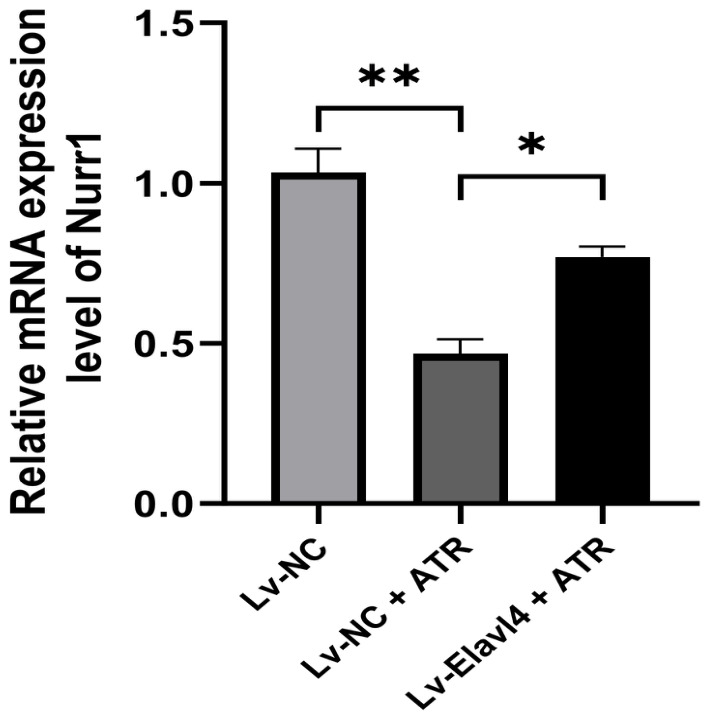
Overexpression of *Elavl4* rescues ATR-induced suppression of *Nurr1*. The relative mRNA expression levels of *Nurr1* among the designated groups. Data are presented as mean ± SD (n = 3). (* *p* < 0.05, ** *p* < 0.01, One-way ANOVA followed by Tukey’s post hoc test).

To definitively establish the functional causality of the *Elavl4*–miR-301a-5p–*Nurr1* regulatory axis, we performed in vitro rescue experiments using a lentiviral expression system. Compared to the vehicle control group, ATR exposure significantly suppressed the mRNA expression of *Nurr1* in SK-N-SH cells (Lv-NC + ATR vs. Lv-NC + Vehicle, *p* < 0.01) (Figure 9). However, prior establishment of stable, lentivirus-mediated *Elavl4* overexpression successfully mitigated this suppressive effect. Specifically, *Nurr1* mRNA levels in the Lv-*Elavl4* + ATR group were significantly restored compared to the Lv-NC + ATR group (*p* < 0.05), returning to near-baseline conditions. These rescue data functionally validate our hypothesis: *Elavl4* acts as a critical competing endogenous RNA (ceRNA) that competitively sponges miR-301a-5p, thereby rescuing and sustaining the expression of its downstream target, *Nurr1*, during ATR-induced neurotoxic stress. Notably, the directional consistency between this in vitro functional rescue and the in vivo expression patterns observed in the rat midbrain reinforces the biological relevance of the Elavl4–miR-301a-5p–Nurr1 axis. This internal concordance suggests that the identified ceRNA network is a pivotal mediator across different biological systems, linking molecular dysregulation to the broader landscape of ATR-induced neurotoxicity.

## 3. Discussion

The present study systematically investigated the neurotoxic effects of early-life atrazine exposure using a rat model, integrating behavioral outcomes, molecular expression profiles, and transcriptomic regulatory network analyses. Our findings demonstrate that atrazine potentially mediates PD-like behavioral impairments and dopaminergic neuronal dysfunction, accompanied by substantial transcriptional disturbances involving neuroinflammatory pathways and non-coding RNA regulatory mechanisms. These results not only reinforce growing concerns regarding the neurodevelopmental toxicity of atrazine but also provide novel mechanistic insights into how environmental toxicants disrupt dopaminergic systems.

### 3.1. Early-Life Atrazine Exposure Potentially Mediates PD-like Behavioral Phenotypes and Dopaminergic Dysfunction

Our behavioral results confirmed that atrazine exposure leads to significant locomotor deficits, anxiety-like behaviors, and impairments in spatial learning and memory. These behavioral abnormalities displayed clear dose–response patterns, with the high-dose ATR group showing more severe deficits. Such phenotypes closely mirror established PD animal models [12].

Correspondingly, qRT-PCR analysis demonstrated classical PD-related molecular signatures, including downregulation of *Th* (tyrosine hydroxylase), *Nurr1*, and altered *α-syn* expression. These changes validate the successful induction of dopaminergic neuronal injury and are consistent with hallmark features reported in PD toxicology.

### 3.2. Th, Slc6a3, and Other Hub Genes Reveal Core Dopaminergic Vulnerabilities Under Atrazine Exposure

Among the hub genes identified from the PPI network, *Th*, *Slc6a3*, and *Nurr1* emerged as central regulatory nodes. These genes collectively govern dopamine synthesis, release, transport, and neuronal integrity:

*Th*, the rate-limiting enzyme in dopamine synthesis, is widely regarded as a “gold-standard” indicator of dopaminergic neuron health [13]. Its significant downregulation aligns with observed motor deficits and suggests compromised dopamine biosynthesis.

For example, in an MPTP (1-methyl-4-phenyl-1,2,3,6-tetrahydropyridine)-induced Parkinson’s disease model, researchers observed a significant reduction in striatal dopamine levels as well as decreased *Th* and *Slc6a3* expression in model animals, directly confirming damage to dopaminergic nerve terminals [14].

Similarly, in studies evaluating compound protective effects, upregulation of *Th* expression is regarded as direct evidence of neuronal preservation. For instance, the flavonoid compound galangin has been demonstrated to mitigate MPTP-induced dopaminergic neuronal damage in mice, with its protective action reflected in significantly elevated *Th* protein expression levels [15].

Dopaminergic neurons are particularly vulnerable to oxidative stress due to their high metabolic activity and the reactive oxygen species generated during dopamine metabolism. As the central enzyme in this pathway, *Th* function is highly context-dependent. Research indicates that free radicals and oxidative stress not only directly damage neurons but also impair *Th* function, further reducing dopamine synthesis and creating a vicious cycle [16].

*Slc6a3*, responsible for dopamine reuptake, was also downregulated. This gene is not only crucial for dopaminergic neurotransmission precision but also serves as an entry point for neurotoxins such as MPP+ [17], explaining why dopaminergic neurons may be particularly susceptible to atrazine.

*Slc6a3* is responsible for reuptaking dopamine from the synaptic cleft back into neurons, serving as a key molecule in regulating the spatiotemporal precision of dopamine signaling and maintaining synaptic microenvironment stability. Its physiological function also makes Slc63 a pathway for certain neurotoxins to enter neurons. The most classic example is MPP+ (a toxic metabolite of MPTP), which is actively transported into dopaminergic neurons via *Slc6a3* as a substrate. It accumulates in mitochondria, inhibits Complex I, and ultimately leads to cellular energy depletion and death [18]. This mechanism explains why MPTP/MPP+ selectively damages dopaminergic neurons. Because *Slc6a3* is specifically expressed on the presynaptic membrane of dopaminergic neurons, its expression levels are highly sensitive to neuronal integrity. Therefore, similar to *Th*, reduced *Slc6a3* expression is widely used as a marker to assess the extent of damage to dopaminergic neuronal terminals [19].

*Nurr1*, a master transcription factor for dopaminergic neuron development and survival, was markedly reduced. Its downregulation is strongly linked to neurodegeneration and is recognized as a potential therapeutic target [20].

Together, these coordinated transcriptional disturbances imply that atrazine simultaneously impairs dopamine synthesis, transport, and cytoprotection—providing a mechanistic explanation for the observed PD-like phenotypes.

### 3.3. Oxidative Stress and Neuroinflammation as Major Contributors to Atrazine-Induced Dopaminergic Injury

Functional enrichment analyses revealed that differentially expressed mRNAs were significantly enriched in: Neuroactive ligand–receptor interaction and Cytokine–cytokine receptor interaction. These pathways are highly relevant to PD pathogenesis.

The Neuroactive ligand–receptor interaction pathway exhibits multiple connections with Parkinson’s disease (PD), mainly involving gene expression regulation, inflammatory responses, gut microbiota effects, and mechanisms of pharmacological interventions.

In a Drosophila PD model, the expression of *α-syn* carrying the A30P mutation upregulates multiple miRNAs (such as dme-*miR-133-3p* and dme-*miR-137-3p*). Among them, *miR-137* exacerbates neurotoxicity by targeting key receptor genes within the Neuroactive ligand–receptor interaction pathway, including the dopamine receptor D2R, the GABA receptor GABA-B-R3, and the NMDA receptor Nmdar2. Experimental evidence has confirmed that *miR-137* binds to the 3′ UTRs of these receptor genes and suppresses their expression, thereby contributing to PD pathogenesis [21].

Integrated analyses of multicenter microarray datasets reveal that differentially expressed genes in the substantia nigra of PD patients are significantly enriched in the Neuroactive ligand–receptor interaction pathway, along with the dopaminergic synapse and calcium signaling pathways. Although gene signatures in peripheral blood are comparatively weaker, the aberrant activation of this pathway in the substantia nigra is highly associated with PD pathology [22].

Loss of the G protein-coupled receptor Gpr35 aggravates neuroinflammation and dopaminergic neuronal injury in MPTP-induced PD models. Mechanistically, Gpr35 modulates gut microbiota (e.g., Lactobacillus) and their metabolites, thereby influencing the Neuroactive ligand–receptor interaction pathway and tryptophan metabolism, ultimately ameliorating PD pathology [23].

Umbilical cord blood–derived exosomes (UCB-Exos) can alleviate motor dysfunction and neuronal damage in PD mice. Transcriptomic analysis showed that UCB-Exos significantly regulated genes associated with the Neuroactive ligand–receptor interaction pathway and dopaminergic synapses, while suppressing the excessive activation of the MAPK signaling pathway [24].

Network analyses reveal shared genetic perturbations between PD and Alzheimer’s disease (AD). Notably, the Neuroactive ligand–receptor interaction pathway is disrupted in both diseases, suggesting its broad role in neurodegenerative disorders [25].

Collectively, these findings highlight the central role of the Neuroactive ligand–receptor interaction pathway in PD pathology and support its potential as a source of novel biomarkers and therapeutic targets.

Activation of the Cytokine–cytokine receptor interaction pathway provides strong evidence that neuroinflammation plays a major role in atrazine-induced neurotoxicity. It is well known that microglial activation leads to the release of pro-inflammatory cytokines such as IL-1β and TNF-α, which, through this pathway, exacerbate oxidative stress and neuronal apoptosis [26]. Notably, Il1b is also included among our hub genes, providing direct transcriptional evidence for the neuroinflammatory mechanism.

Studies have shown that the traditional Chinese medicine formula DiHuangYin exerts therapeutic effects by suppressing the IL-17 signaling pathway. In MPTP-induced PD mouse models, this formula reduces the number of splenic Th17 cells and serum IL-17A levels, thereby mitigating peripheral immune inflammation and reducing its impact on the central nervous system, ultimately protecting dopaminergic neurons in the substantia nigra pars compacta [27].

Network pharmacology analysis of Paeonia lactiflora (white peony root) indicates that the TNF signaling pathway is one of its core mechanisms in treating PD. TNF-α is a classic pro-inflammatory cytokine whose binding to its receptor strongly activates downstream pathways such as NF-κB, exacerbating neuroinflammation and neuronal apoptosis. This process represents a canonical activation route within the Cytokine–cytokine receptor interaction pathway [28].

Research has confirmed that the G protein-coupled receptor Gpr35 is a key regulator of neuroinflammation in PD. Loss of Gpr35 exacerbates neuroinflammation and microglial activation in MPTP models, whereas its agonists suppress inflammatory responses and neuronal apoptosis. Although not all cytokines were listed in the study, activated microglia typically release large amounts of pro-inflammatory cytokines (e.g., TNF-α and IL-1β), which engage with their receptors to form a network of inflammatory injury [23].

Umbilical cord blood-derived exosomes (UCB-Exos) have also been shown to significantly improve pathological symptoms in PD mouse models. Their mechanism includes inhibiting excessive MAPK phosphorylation, a critical downstream signaling event of many cytokine receptors. The anti-inflammatory effect of these exosomes suggests they may indirectly modulate inflammatory output through the cytokine–receptor interaction axis [24].

Although direct studies on the Cytokine–cytokine receptor interaction pathway are limited, strong indirect evidence indicates that many cytokines within this pathway (e.g., IL-17 and TNF-α) and their receptors play central roles in PD-associated neuroinflammation. The neuroinflammatory responses in PD are largely driven by cytokines derived from microglia and peripheral immune cells interacting through this pathway.

### 3.4. Significance of the Early-Life Exposure Window

In this study, the selected early-life atrazine exposure window and dosing regimen were based on environmental relevance and the heightened susceptibility of developmental neurotoxicity. First, in terms of dosage, we employed exposure concentrations of 1 mg/kg/day (low dose) and 10 mg/kg/day (high dose), both well below levels that induce acute systemic toxicity, yet capable of mimicking realistic long-term environmental exposure through drinking water and the food chain. Due to bioaccumulation, actual organismal burdens may approach the lower dose range used in this study, supporting its environmental relevance and mechanistic specificity.

While the general population is typically exposed to trace levels of atrazine via regulated drinking water, significantly higher exposure scenarios exist. Agricultural runoff, contaminated local well water, and occupational handling in agricultural communities can lead to substantial bioaccumulation. The dosing regimen utilized in our rodent model (1 and 10 mg/kg/day) effectively simulates these amplified, cumulative toxicological burdens experienced by vulnerable populations, underscoring the public health relevance of this early-life exposure window.

Second, the exposure window—from gestation to weaning—has strong toxicological justification. This life stage represents a “critical window” of central nervous system development, characterized by neurogenesis, neuronal migration, synaptogenesis, and maturation of the dopaminergic system. During this period, the blood–brain barrier is not fully developed, rendering the brain highly susceptible to exogenous chemicals [29]. Evidence indicates that early-life exposure to environmental toxicants can induce persistent alterations in neural structure and function via developmental programming, increasing susceptibility to neurodegenerative diseases in adulthood—a core concept of the Developmental Origins of Health and Disease (DOHaD) hypothesis [30]. Our study focuses on this critical window to elucidate how atrazine disrupts the establishment and homeostasis of the dopaminergic network through developmental programming, thereby providing forward-looking experimental evidence for environment-driven neurodevelopmental disturbances and long-term PD risk.

One of the most significant findings of this study is the construction of a ceRNA regulatory network centered on the transcription factor *Nurr1* (*Elavl4*–miR-301a-5p–*Nurr1*). *Nurr1* plays an essential role in dopaminergic neuron development and survival, and its downregulation is closely associated with PD onset. This study proposes that the lncRNA *Elavl4* may indirectly regulate *Nurr1* expression by sponging miR-301a-5p, and that atrazine exposure disturbs this regulatory axis, thereby contributing to dopaminergic neuronal injury. This discovery not only deepens our understanding of the post-transcriptional mechanisms underlying atrazine neurotoxicity but also provides a promising direction for early therapeutic intervention.

### 3.5. The Elavl4/miR-301a-5p/Nurr1 Regulatory Axis: A Novel Perspective on Dopaminergic Neuron Injury Induced by the Environmental Toxicant ATR

First, the dual-luciferase reporter assay addressed the question of whether binding occurs. The results demonstrated that *miR-301a-5p* specifically recognizes sequences within *Elavl4* and the *Nurr1* 3′UTR, and that this interaction depends on seed-sequence complementarity [31].

Second, the RIP assay clarified where this interaction takes place. Ago2 serves as the functional core of the ceRNA regulatory network [32]. Our findings confirmed that *Elavl4*, *miR-301a-5p*, and *Nurr1* are co-enriched within the RISC under physiological cellular conditions, a prerequisite for ceRNA-mediated post-transcriptional regulation.

Finally, the RNA pull-down assay addressed whether the interaction is direct [33]. Using a biotin-labeled capture strategy, we directly isolated complexes containing *Elavl4* and *miR-301a-5p*, providing biochemical evidence of their physical association.

Collectively, these results support the establishment of a toxicological regulatory model. Under physiological conditions in dopaminergic neurons, lncRNA *Elavl4* is abundantly expressed and functions as a molecular sponge, sequestering *miR-301a-5p* and thereby protecting *Nurr1* from miRNA-mediated repression. This mechanism contributes to the maintenance of dopaminergic neuronal integrity. However, upon exposure to the environmental toxicant ATR, *Elavl4* expression is downregulated, as indicated by our transcriptomic analysis. The reduced sponge capacity results in increased availability of *miR-301a-5p*, which in turn enhances repression of *Nurr1*, ultimately leading to dopaminergic neuronal dysfunction and Parkinsonian-like pathology [34].

Given that *Nurr1* is widely recognized as a promising therapeutic target for Parkinson’s disease [35], elucidating its upstream regulatory mechanisms is of critical importance. The present study not only uncovers a novel post-transcriptional pathway underlying ATR-induced neurotoxicity but also suggests that modulation of *Elavl4* or *miR-301a-5p* may represent a potential strategy to restore *Nurr1* expression and mitigate dopaminergic neurodegeneration.

**Figure 10:**
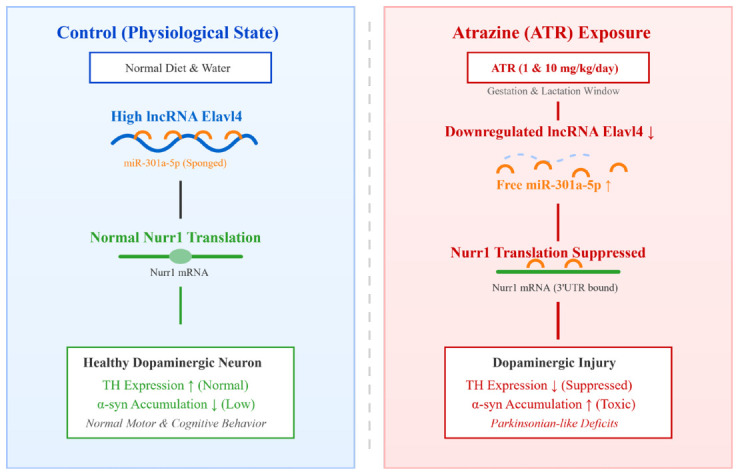
Schematic model of the proposed ceRNA regulatory mechanism underlying ATR-induced dopaminergic neurotoxicity. Under physiological conditions (**left panel**), abundant lncRNA *Elavl4* acts as a molecular sponge to sequester miR-301a-5p, permitting normal *Nurr1* translation, which maintains healthy TH expression and supports normal motor and cognitive functions. Conversely, gestational and lactational exposure to atrazine (**right panel**) significantly downregulates *Elavl4*, leading to the release of free miR-301a-5p. This accumulated miRNA directly binds to the 3′UTR of *Nurr1* mRNA and suppresses its translation. The resulting Nurr1 deficiency triggers decreased TH expression and aberrant α-syn accumulation, ultimately culminating in dopaminergic neuronal injury and Parkinsonian-like behavioral deficits.

To clearly illustrate these findings, we have proposed a schematic model summarizing the ATR-induced ceRNA regulatory network and its downstream pathological consequences (Figure 10). 

### 3.6. Mechanistic Implications and Limitations

Although our in vitro rescue experiments establish a clear functional regulatory loop, we recognize the inherent complexity of the in vivo neurodevelopmental environment. The transition from molecular signaling to systemic behavioral deficits involves multi-synaptic integrations that cannot be fully captured in cell-based models. Therefore, while our data identifies the Elavl4–miR-301a-5p–Nurr1 axis as a primary mechanistic driver, we consider it a key node within a larger, more complex neurotoxicological network. Future studies utilizing site-specific in vivo gene interference will be pivotal to further refine this therapeutic targeting.

Importantly, while our in vitro rescue experiments establish a functional ceRNA axis at the cellular level, the lack of in vivo genetic intervention (e.g., stereotaxic delivery of Elavl4-overexpressing viral vectors) means the direct causal link between this network and the observed behavioral deficits in rats remains to be definitively established. Currently, this axis represents a strongly correlated molecular pathway rather than a proven in vivo driver of the phenotype.

Despite these promising findings, several limitations of the present study must be acknowledged. First, to address the inherent limitations of undifferentiated neuroblastoma cells, we implemented retinoic acid (RA)-induced differentiation of SK-N-SH cells into a post-mitotic, dopaminergic-like phenotype, which significantly improves the physiological relevance of our in vitro model. Nevertheless, future validation in primary midbrain cultures or iPSC-derived neurons will further solidify these findings. Second, validation experiments were conducted in the human SK-N-SH cell line, whereas the transcriptomic discovery phase utilized rat midbrain tissue. Although our bioinformatic analyses indicate high evolutionary conservation of the predicted miR-301a-5p binding seed sequences within the 3′UTRs of both human and rat *Nurr1* and *Elavl4*, species-specific differences in broader ceRNA network architecture cannot be entirely ruled out. Future studies utilizing primary rat midbrain dopaminergic neurons are warranted to fully bridge this cross-species gap. Third, while our lentivirus-mediated rescue experiments provide robust in vitro evidence for the functional causality of the *Elavl4*–miR-301a-5p–*Nurr1* axis, in vivo rescue interventions are ultimately necessary to definitively confirm this causal mechanism within the complex microenvironment of the intact brain. Translating these findings into therapeutic interventions will require targeted in vivo proof-of-concept models. Future strategies should focus on precision delivery systems, such as utilizing Adeno-Associated Virus (AAV) vectors for midbrain-specific overexpression of *Elavl4*, or employing nanoparticle-encapsulated antisense oligonucleotides (ASOs) to selectively antagonize miR-301a-5p. Evaluating whether these targeted in vivo interventions can successfully reverse ATR-induced motor and cognitive deficits will be the critical next step in determining their true therapeutic viability. Furthermore, our current experimental design prioritized distinct cohort allocation for behavioral tracking and molecular tissue analysis to minimize the confounding effects of testing-induced stress on transcriptomic profiles. Consequently, we were unable to perform direct 1-to-1 correlational analyses between specific behavioral deficit scores and individual *Nurr1* or miR-301a-5p expression levels. Future studies incorporating paired molecular-behavioral tracking within the same individuals are essential to further validate the physiological relevance of this ceRNA network.

Lastly, human sporadic Parkinson’s disease is characterized by a highly complex, multifactorial etiology. Extrapolating these developmental rat-model findings to human pathogenesis should be interpreted with caution, positioning this axis as a robust hypothesis-generating target rather than a definitive clinical mechanism.

## 4. Materials and Methods

### 4.1. Animal Model and Ethical Approval

Male and female Sprague–Dawley (SD) rats (6–8 weeks old) were purchased from Henan Scibest Biotechnology Co., Ltd., China (Henan, China), an accredited laboratory animal supplier (production license number: SCXK(Yu)2020~0005). All animals were housed under specific pathogen-free (SPF) conditions at Guilin Medical University. Environmental conditions were maintained at 21–25 °C, 40–60% humidity, with a 12 h light/dark cycle. Standard chow and autoclaved water were provided ad libitum.

An a priori power analysis was performed using G*Power software (v3.1) before the experiment. Based on an assumed large effect size (f = 0.4), an α level of 0.05, and a statistical power of 0.80, at least 8 litters per group were required.

After mating, pregnant dams were randomly assigned to three groups (n = 8 dams per group) using a computer-generated randomization sequence: a control group, a low-dose ATR exposure group, and a high-dose ATR exposure group. Atrazine exposure was administered throughout gestation and lactation. Offspring were subjected to behavioral testing at postnatal day 21 (PND21) and sacrificed for tissue collection at PND28. To minimize litter effects, the litter, rather than the individual pup, was regarded as the experimental unit.

### 4.2. Atrazine Exposure Protocol

Atrazine (ATR, CAS: 1912-24-9; Admas-beta, purity ≥97%) was suspended in 3% (*w*/*v*) starch paste dissolved in ultra-pure distilled water (Nanjing Ganzhiyuan Food Co., Ltd., Nanjing, China). Fresh suspensions were prepared daily to ensure stability. Based on WHO-recommended exposure models [5], pregnant rats received either low-dose ATR (1 mg/kg/day) or high-dose ATR (10 mg/kg/day). It is important to contextualize these doses against estimated human environmental exposures. While general population exposure via drinking water is typically strictly regulated at trace levels (e.g., the US EPA Maximum Contaminant Level for atrazine is 3 μg/L), the doses utilized in our rodent model are substantially higher. However, they are specifically designed and widely utilized in developmental neurotoxicity studies to effectively simulate the accelerated, cumulative toxicological burden experienced by vulnerable populations in high-exposure occupational agricultural settings, particularly during the highly sensitive early-life developmental window. Control animals received an equal volume of the vehicle (1 mL per 100 g body weight). Oral gavage was performed daily between 08:00 and 10:00 using a stainless-steel feeding needle with a ball tip to minimize esophageal injury.

### 4.3. Tissue Collection and Behavioral Assessment

To rigorously control for litter effects and strictly avoid pseudoreplication, our sampling strategy defined the independent litter as the experimental unit. Specifically, one offspring was randomly selected from each of the 8 independent litters per treatment group. Furthermore, to account for sex as a biological variable, we maintained a strict 1:1 sex ratio within each group (n = 4 males and 4 females, yielding a final sample size of 8 independent biological replicates per group). After weaning, these selected offspring underwent assessments of locomotor and cognitive function using the open field test, elevated plus maze, and Morris water maze. Following behavioral testing, rats were anesthetized with sodium pentobarbital and euthanized. Midbrain tissues were collected and separated: the left hemisphere was flash-frozen in liquid nitrogen for transcriptome sequencing, while the right hemisphere was fixed in 4% paraformaldehyde for histological analysis. All behavioral testing and subsequent data analyses were performed by investigators blinded to the treatment group assignments.

### 4.4. Whole-Transcriptome Sequencing

Total RNA was extracted from midbrain tissue using TRIzol reagent. RNA integrity (RIN ≥ 7.0) was confirmed with an Agilent 2100 Bioanalyzer. Libraries were prepared using the Illumina Stranded Total RNA Prep Kit and sequenced on a NovaSeq platform (PE150). Raw data were subjected to quality control with FastQC and filtered using Trimmomatic. Reads were aligned to the rat reference genome (rn6) using HISAT2. Differential expression analysis was performed in DESeq2 (thresholds: |log_2_FC| > 1.5 and adjusted *p* < 0.05).

### 4.5. Protein–Protein Interaction (PPI) Network and Hub Gene Identification

Differentially expressed mRNAs (DEMs) were uploaded to the STRING database (organism: Rattus norvegicus) to obtain protein–protein interaction data. Cytoscape was used to visualize the PPI network. Hub genes were identified using the CytoHubba plugin based on four algorithms (Degree, MCC, Closeness, and Betweenness), and the top 10 hub genes were selected.

### 4.6. Regulatory Network Construction

Differentially expressed lncRNAs, miRNAs, and mRNAs were analyzed using StarBase, lncBase, TargetScan, and miRWalk to predict interactions. Overlapping predicted targets and experimentally identified differentially expressed genes were integrated to construct lncRNA–mRNA, miRNA–mRNA co-expression networks, and the final lncRNA–miRNA–mRNA ceRNA regulatory network.

### 4.7. Quantitative Real-Time PCR (qRT-PCR) Validation

To validate transcriptome results, qRT-PCR was performed on key PD marker genes and hub genes using specific primers. Expression trends were compared with sequencing data to confirm reliability.

### 4.8. Cell Culture and Model Establishment

The human neuroblastoma cell line SK-N-SH was obtained from the Cell Bank of the Chinese Academy of Sciences. Cells were initially cultured in DMEM/F12 medium supplemented with 10% fetal bovine serum (FBS) and 1% penicillin-streptomycin under standard conditions (37 °C, 5% CO_2_ in a humidified incubator). Cells in the logarithmic growth phase were used for subsequent experimental preparation.

To establish a physiologically relevant model of mature, post-mitotic dopaminergic neurons, SK-N-SH cells were subjected to differentiation prior to toxicant exposure. Specifically, the culture medium was replaced with a low-serum differentiation medium containing 10 μM all-trans-retinoic acid (RA; Sigma-Aldrich, St. Louis, MO, USA). The medium was refreshed every 48 h for 7 days until robust neurite outgrowth and a post-mitotic neural network morphology were observed.

Following successful differentiation, to establish the atrazine (ATR)-induced neurotoxicity model, the fully differentiated cells were treated with ATR at a final concentration of 50 μM for 24 h to simulate environmental toxin exposure in mature dopaminergic neurons. Control cells received an equal volume of the vehicle (dimethyl sulfoxide, DMSO).

### 4.9. Western Blotting and Immunohistochemistry (IHC)

Western Blotting: Total protein was extracted from rat midbrain tissues using RIPA lysis buffer supplemented with protease and phosphatase inhibitors. Protein concentrations were determined using a BCA Protein Assay Kit. Equal amounts of protein extracts (20 μg per lane) were separated by SDS-PAGE and electrophoretically transferred onto PVDF membranes. The membranes were blocked with 5% non-fat dry milk in Tris-buffered saline containing 0.1% Tween 20 (TBST) for 2 h at room temperature. Subsequently, the membranes were incubated overnight at 4 °C with the following primary antibodies: anti-Tyrosine Hydroxylase (TH) (1:1000, Proteintech, Rosemont, IL, USA), anti-alpha Synuclein (1:2000, Proteintech, USA), and anti-GAPDH (1:5000, Proteintech, USA), which served as an internal loading control. After three washes with TBST, the membranes were incubated with HRP-conjugated Goat Anti-Rabbit Recombinant Secondary Antibody (1:5000, Proteintech, USA) for 1 h at room temperature. Protein bands were visualized using an enhanced chemiluminescence (ECL) detection kit, and the relative optical density of the bands was quantified using ImageJ 1.54p software.

Immunohistochemistry: To assess dopaminergic neuronal damage morphologically, brain tissues encompassing the substantia nigra pars compacta (SNpc) were fixed in 4% paraformaldehyde, embedded in paraffin, and cut into 5 μm thick coronal sections. Following deparaffinization in xylene and rehydration through a graded ethanol series, heat-induced antigen retrieval was performed in citrate buffer (pH 6.0). Endogenous peroxidase activity was quenched with 3% H_2_O_2_, and nonspecific binding was blocked with 5% bovine serum albumin (BSA) for 1 h at room temperature. The sections were then incubated overnight at 4 °C with the anti-TH primary antibody (1:200 dilution, Proteintech, USA). After thorough washing in PBS, the sections were incubated with an HRP-conjugated secondary antibody for 1 h at room temperature. Immunoreactivity was visualized using a 3,3′-diaminobenzidine (DAB) substrate kit, which yields a brown precipitate at the antigen site. The stained sections were observed, and images were captured under a light microscope.

### 4.10. Dual-Luciferase Reporter Assay

To validate the direct targeting relationship between miR-301a-5p and *Elavl4* or *Nurr1*, a Dual-Luciferase Reporter Assay was performed as follows:

#### 4.10.1. Plasmid Construction

Putative miR-301a-5p binding sites were predicted using TargetScan and miRWalk. DNA fragments containing the predicted binding sequences from *Elavl4* and the *Nurr1* 3′ untranslated region (3′UTR) (wild-type, WT) were amplified and cloned into the pmirGLO luciferase reporter vector. Mutant constructs (MUT) harboring site-directed mutations within the predicted binding regions were generated using a site-directed mutagenesis kit.

#### 4.10.2. Cell Transfection

HEK293T cells were seeded into 96-well plates. When cell confluence reached approximately 70%, reporter plasmids were co-transfected with miR-301a-5p mimics or negative control (NC) mimics using Lipofectamine 3000 (Invitrogen, Carlsbad, CA, USA) according to the manufacturer’s instructions.

#### 4.10.3. Luciferase Activity Measurement

Forty-eight hours post-transfection, luciferase activity was measured using a Dual-Luciferase Reporter Assay Kit (Promega, Madison, WI, USA). Firefly luciferase activity was normalized to Renilla luciferase activity, and relative luciferase activity was calculated accordingly.

### 4.11. RNA Immunoprecipitation (RIP) Assay

To determine whether *Elavl4*, miR-301a-5p, and *Nurr1* are present within the RNA-induced silencing complex (RISC), RIP assays were performed.

#### 4.11.1. Cell Lysis

SK-N-SH cells were harvested and lysed in RIP lysis buffer supplemented with RNase inhibitors and protease inhibitors.

#### 4.11.2. Immunoprecipitation

A portion of the lysate was reserved as input control. The remaining lysate was incubated overnight at 4 °C with magnetic beads conjugated to anti-Ago2 antibody (Millipore, Burlington, MA, USA) or normal IgG as a negative control. Ago2 is the core component of the RISC responsible for miRNA-mediated gene silencing.

#### 4.11.3. RNA Isolation and Detection

Following protein digestion, co-immunoprecipitated RNA was extracted and subjected to quantitative real-time PCR (qRT-PCR) to determine the enrichment of *Elavl4*, miR-301a-5p, and *Nurr1*. Results were expressed as a percentage of input (% Input).

### 4.12. RNA Pull-Down Assay

To further confirm the direct physical interaction between *Elavl4* and miR-301a-5p, RNA pull-down assays were conducted.

#### 4.12.1. Probe Preparation

Biotin-labeled antisense *Elavl4* probes (Bio-*Elavl4*) and corresponding negative control probes (Bio-NC) were synthesized. Alternatively, biotin-labeled miR-301a-5p mimics (Bio-miR-301a-5p) were used.

#### 4.12.2. RNA Capture

Biotin-labeled RNA or probes were transfected into SK-N-SH cells or incubated with cell lysates under appropriate conditions.

#### 4.12.3. Magnetic Bead Enrichment

Streptavidin-coated magnetic beads were added to capture the biotin-labeled RNA–protein complexes.

#### 4.12.4. Analysis of Bound RNA

After extensive washing, bound RNA was extracted and analyzed by qRT-PCR to detect enrichment of interacting molecules (e.g., miR-301a-5p enriched by Bio-*Elavl4*, or *Nurr1*/*Elavl4* enriched by Bio-miR-301a-5p).

### 4.13. Lentiviral Infection and Rescue Experiments

To overexpress *Elavl4*, recombinant lentiviruses carrying the full-length *Elavl4* sequence (Lv-*Elavl4*) and negative control lentiviruses (Lv-NC) were constructed and packaged by GeneChem Co., Ltd. (Shanghai, China) using the GV513 lentiviral vector (Ubc-MCS-CBh-gcGFP-IRES-puromycin). SK-N-SH cells were seeded into 6-well plates and grown to approximately 50–60% confluence. The cells were then infected with the respective lentiviruses at an appropriate multiplicity of infection (MOI). To enhance the lentiviral transduction efficiency while minimizing cytotoxicity, HitransG P infection enhancer (GeneChem, Daejeon, Republic of Korea) was added to the culture medium according to the manufacturer’s instructions. Successful infection was visually monitored by observing the expression of Green Fluorescent Protein (GFP) under a fluorescence microscope. After stable lentiviral transduction was established (typically 72 h post-infection), the cells were exposed to 50 μM ATR for 24 h. Following the treatments, total RNA was extracted, and qRT-PCR was performed to evaluate the restorative effect of *Elavl4* overexpression on *Nurr1* mRNA levels.

### 4.14. Statistical Analysis

All in vitro experiments were performed using at least three independent biological replicates, each with three technical replicates. Animal data represents independent litters (n = 8 per group, balanced by sex). Data are presented as mean ± standard deviation (SD). Statistical analyses were conducted using GraphPad Prism 10.0. For multiple testing, False Discovery Rate (FDR) correction (Benjamini–Hochberg procedure) was applied to transcriptomic and qRT-PCR data. Behavioral multiple comparisons were evaluated using one-way ANOVA followed by Tukey’s post hoc test. To convey the magnitude of biological differences, effect sizes were calculated and reported as eta-squared (η^2^) for multiple group comparisons (ANOVA). A corrected *p* value < 0.05 was considered statistically significant. Detailed descriptive statistics, *p*-values, and corresponding effect sizes for all key endpoints are provided in Supplementary Table S1.

## 5. Conclusions

Gestational and lactational ATR exposure induced behavioral abnormalities and dopaminergic injury in rat offspring. Whole-transcriptome analysis combined with experimental validation suggests a potential association between the Elavl4/miR-301a-5p/Nurr1 ceRNA axis and ATR-induced dopaminergic injury, providing insight into the post-transcriptional mechanisms underlying developmental neurotoxicity. Further in vivo studies are needed to confirm this mechanism in the physiological brain environment.

## Data Availability

The raw and processed whole-transcriptome sequencing data generated in this study have been deposited in the NCBI Sequence Read Archive (SRA) under BioProject accession number PRJNA1440964. These data will be publicly available upon publication of this article.

## Supplementary Materials

The following supporting information can be downloaded at: https://www.mdpi.com/article/10.3390/ijms27093818/s1.
