# Supplementary material for: Gestational and Lactational Atrazine Exposure Potentially Mediates Behavioral and Dopaminergic Alterations in Rat Offspring: Insights into Nurr1-Related ceRNA Regulation
Source: Int J Mol Sci. 2026 Apr 25;27(9):3818. doi: 10.3390/ijms27093818 (PMC13163254; doi:10.3390/ijms27093818)
Supplement: Supplementary file 1 [file ijms-27-03818-s001.zip › ijms-4208904-supplementary.pdf]

Supplementary Table S1

Indicator	Con	L-ATR	H-ATR	n/group	P	η^2	Magnitude
miR-301a-5p	1.001±0.062	1.695±0.108	2.682±0.175	3	< 0.0001	0.979	Large
Elavl4	1.002±0.080	0.624±0.010	0.434±0.025	3	< 0.0001	0.972	Large
Nurr1	1.005±0.121	0.702 ± 0.089	0.268±0.056	3	0.0002	0.941	Large
TH（mRNA）	1.008±0.156	0.383 ± 0.066	0.226±0.023	3	0.0002	0.946	Large
Body Weight	374.34±17.00	348.01±10.11	331.36±6.89	8	< 0.0001	0.71	Large
OFT Time	15.61 ± 4.55	8.19 ± 4.41	4.06 ± 3.40	8	< 0.0001	0.602	Large
OFT Cross	6.75 ± 3.77	3.50 ± 1.60	0.88 ± 0.99	8	0.0004	0.527	Large
EPM	34.21 ± 5.16	22.48 ± 4.92	11.90 ± 4.20	8	< 0.0001	0.806	Large
MWM Cross	3.63 ± 1.06	1.88 ± 0.64	0.75 ± 0.71	8	< 0.0001	0.702	Large
MWM Distance	17.07 ± 2.32	13.90 ± 2.44	10.98 ± 1.38	8	< 0.0001	0.616	Large
MWM Time	1.71 ± 0.51	1.14 ± 0.07	0.36 ± 0.09	8	< 0.0001	0.79	Large
